# A Snapshot, Using a Multi-Omic Approach, of the Metabolic Cross-Talk and the Dynamics of the Resident Microbiota in Ripening Cheese Inoculated with *Listeria innocua*

**DOI:** 10.3390/foods13121912

**Published:** 2024-06-18

**Authors:** Alessandra Tata, Andrea Massaro, Brunella Miano, Sara Petrin, Pietro Antonelli, Arianna Peruzzo, Alessandra Pezzuto, Michela Favretti, Marco Bragolusi, Carmela Zacometti, Carmen Losasso, Roberto Piro

**Affiliations:** 1 Laboratorio di Chimica Sperimentale, Istituto Zooprofilattico Sperimentale delle Venezie, Viale Fiume 78, 36100 Vicenza, Italy; amassaro@izsvenezie.it (A.M.); bmiano@arpae.it (B.M.); mbragolusi@izsvenezie.it (M.B.); czacometti@izsvenezie.it (C.Z.); rpiro@izsvenezie.it (R.P.); 2 Laboratory of Microbial Ecology and Genomics, Istituto Zooprofilattico Sperimentale delle Venezie, Viale dell’Università, 35020 Legnaro, Italy; spetrin@izsvenezie.it (S.P.); pantonelli@izsvenezie.it (P.A.); aperuzzo@izsvenezie.it (A.P.); closasso@izsvenezie.it (C.L.); 3 PhD National Programme in One Health Approaches to Infectious Diseases and Life Science Research, Department of Public Health, Experimental and Forensic Medicine, University of Pavia, 27100 Pavia, Italy; 4 Laboratory of Hygiene and Safety of the Food Chain, Istituto Zooprofilattico Sperimentale delle Venezie, Viale dell’Università, 35020 Legnaro, Italy; apezzuto@izsvenezie.it (A.P.); mfavretti@izsvenezie.it (M.F.)

**Keywords:** *Listeria*, direct analysis in real time high-resolution mass spectrometry, data fusion, ambient mass spectrometry, microbial cross-talk, microbial networking, lactic acid bacteria

## Abstract

Raw milk cheeses harbor complex microbial communities. Some of these microorganisms are technologically essential, but undesirable microorganisms can also be present. While most of the microbial dynamics and cross-talking studies involving interaction between food-derived bacteria have been carried out on agar plates in laboratory-controlled conditions, the present study evaluated the modulation of the resident microbiota and the changes of metabolite production directly in ripening raw milk cheese inoculated with *Listeria innocua* strains. Using a proxy of the pathogenic *Listeria monocytogenes*, we aimed to establish the key microbiota players and chemical signals that characterize Latteria raw milk cheese over 60 days of ripening time. The microbiota of both the control and *Listeria*-inoculated cheeses was analyzed using 16S rRNA targeted amplicon sequencing, while direct analysis in real time mass spectrometry (DART-HRMS) was applied to investigate the differences in the metabolic profiles of the cheeses. The diversity analysis showed the same microbial diversity trend in both the control cheese and the inoculated cheese, while the taxonomic analysis highlighted the most representative genera of bacteria in both the control and inoculated cheese: *Lactobacillus* and *Streptococcus*. On the other hand, the metabolic fingerprints revealed that the complex interactions between resident microbiota and *L. innocua* were governed by continuously changing chemical signals. Changes in the amounts of small organic acids, hydroxyl fatty acids, and antimicrobial compounds, including pyroglutamic acid, hydroxy-isocaproic acid, malic acid, phenyllactic acid, and lactic acid, were observed over time in the *L. innocua*-inoculated cheese. In cheese that was inoculated with *L. innocua*, *Streptococcus* was significantly correlated with the volatile compounds carboxylbenzaldheyde and cyclohexanecarboxylic acid, while *Lactobacillus* was positively correlated with some volatile and flavor compounds (cyclohexanecarboxylic acid, pyroxidal acid, aminobenzoic acid, and vanillic acid). Therefore, we determined the metabolic markers that characterize a raw milk cheese inoculated with *L. innocua*, the changes in these markers with the ripening time, and the positive correlation of flavor and volatile compounds with the resident microbiota. This multi-omics approach could suggest innovative food safety strategies based on the enhanced management of undesirable microorganisms by means of strain selection in raw matrices and the addition of specific antimicrobial metabolites to prevent the growth of undesirable microorganisms.

## 1. Introduction

Raw milk cheeses harbor complex microbial communities composed of bacteria and fungi. Some of these microorganisms are technologically essential and invest food with desirable sensorial characteristics. However, undesirable microorganisms can also be present; these can reduce the quality of production (spoilage microorganisms) or even negatively affect their safety (pathogens) and, thus, affect public health. The current methods for monitoring and controlling cheese safety focus on compliance with process hygiene and food safety criteria. Specifically, food business operators (FBOs) have to implement general requirements to achieve suitable hygiene of foodstuffs and guarantee the absence (or reduced presence) of undesirable microorganisms in processed and unprocessed foodstuffs intended for human consumption [1].

However, these methods have traditionally been devoted only to the detection and identification of contaminant microorganisms in raw milk cheese matrices, without any consideration of the effect either of constant and fluctuating bacteria (in terms of numbers and types) impacting physico-chemical parameters or of their metabolic interactions with the resident microbiota. The impact of the resident microbiota on the growth dynamics of undesirable bacteria might be significant. The dominating microbiota can inhibit the growth of non-resident microorganisms by means of metabolic response, and in the same way, they inhibit their own growth, depending on microbial population density, thus impacting the safety, aroma, and taste of products. Additionally, the stressful conditions of some food (eco)systems, particularly lactic fermentations, can induce diverse microbial growth dynamics strongly linked to the resident bacterial strains’ specificity and metabolism. Specifically, the resident microbiota produce antimicrobial compounds, including small organic molecules, lipids, biosurfactants, bacteriocins, and bacteriocin-like peptides against the undesirable microorganisms [2,3].

Among the undesirable microorganisms in cheese, one potential target is *Listeria monocytogenes*. This is a main causative agent of food poisoning [4,5], and the number of listeriosis cases has been progressively increasing in recent years [6]. To develop novel and effective methods for controlling *L. monocytogenes* in cheese production, a better understanding is needed regarding the metabolic mechanisms of interactions between *Listeria* spp. and the resident microbiota in raw milk cheese.

The complete understanding of bacterial metabolism capable of inhibiting pathogen growth has been the aim of several in vitro studies [7,8,9,10]. Recently, Fuochi et al. [11] detected and quantified *Lactobacillus* spp. metabolites in supernatants of co-cultures of *Legionella* and *Lactobacillus* spp., providing a panel of compounds involved in their interaction. Other studies identified antifungal metabolites produced by *Lactobacillus* spp. against *Penicillium* and *Aspergillum* species [12,13]. Moreover, co-cultures of specific lactic and propionic acid bacteria have been shown to have an antagonistic effect against yeast and molds in dairy systems [14,15,16,17,18].

Most microbial networking studies have been carried out on agar plates in laboratory-controlled conditions, but it is known that the food matrix can affect the bacteria growth dynamics and metabolite production [19,20,21]. On the other hand, the metabolic behavior of *Lactobacillus* spp. in one cheese has been investigated by means of traditional analytical techniques [14], but no detailed description of the released compounds has been reported while interacting with pathogens [16,17,20,22,23,24,25,26]. Recently, Tzora et al. [27] established differences in the molecular signaling (also called cross-talk) exchanged between resident microbiota in Kefalograviera cheese after the feeding system of the dairy sheep was changed. On the other hand, Shang et al. [28], by correlating metataxonomic and metabolomic data, studied the dynamics of microbial communities, the changes in flavor, and the physico-chemical properties of pickled chayote during an industrial-scale natural fermentation.

**Figure 1:**
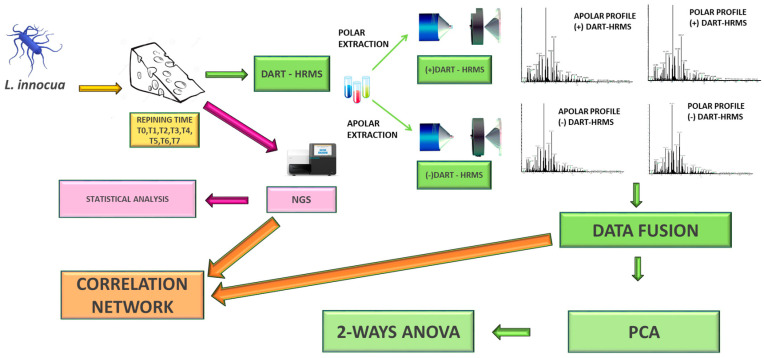
Experimental and analytical workflow of the study. *Listeria innocua*-inoculated cheese and control cheese (i.e., uninoculated cheese) were extracted by two different solvents and analyzed by direct analysis in real time high-resolution mass spectrometry (DART-HRMS) in positive and negative ion mode to tease out metabolomic signatures by statistical analysis (green boxes and arrows). The same samples were also analyzed by next-generation sequencing (NGS) to retrieve metataxonomic differences (pink box and arrow). Metabolomic and metataxonomic data were then correlated by correlation network (orange box and arrow).

Modern molecular tools provide the opportunity for high-resolution analysis of fermentation systems, supported by the growing quality and availability of public sequence data and bioinformatics tools. Next-generation sequencing (NGS), in particular, has reshaped the way to approach microbial ecology, promising cutting-edge discoveries in the field of food microbiology [29]. In addition, mass spectrometry (MS)-based approaches have revolutionized microbiology in the last decade [30,31,32]. Ambient MS (AMS) was applied to monitor metabolite production of live microbial colonies from diverse antagonistic microbial genera in bacterial [33,34,35,36,37,38,39] and fungal co-cultures [40] on agar systems. Among AMS techniques, direct analysis in real time coupled with high-resolution mass spectrometry (DART-HRMS) has proven to be an emerging tool for microbial identification [41,42,43,44], and analysis of fatty acid methyl ester (FAME) ions from whole microbial communities has revealed metabolomics of dairy products [45,46,47]. In this work, the cross-talk of the resident bacteria in cheese inoculated with *L. innocua* strains, as it is widely considered the primary non-pathogenic surrogate of *L. monocytogenes* with the greatest margin of safety [48], was assessed over time by a multi-omics approach using DART-HRMS and NGS coupled with a low-level data fusion statistical strategy with a multi-omics approach (Figure 1).

The overarching goal of this study is the determination of (i) the microbial dynamics of the resident microbiota and (ii) the metabolic changes in ripening cheeses inoculated with *Listeria innocua* strains.

In other words, we aim to define the key microbiota players and/or metabolites that were characteristic of the control cheese and *L. innocua*-inoculated cheese, as well as the correlation network between microbial and metabolic profiles in *L. innocua*-inoculated cheeses.

## 2. Materials and Methods

### 2.1. Cheese Production

For this study, Latteria cheese, a typical Italian cheese made from raw cow milk, was used. The cheesemaking was carried out in an experimental dairy factory set up at a school campus in Cividale del Friuli (UD), in the northeast of Italy, following the traditional processing procedure for Latteria raw milk cheese. The cheesemaking process took two days, and it was carried out in June 2017. The selected starter Lyofast SLH 073 (Sacco System, Cadorago, Italy), consisting of a freeze-dried culture of selected strains of *Streptococcus thermophilus*, *Lactobacillus helveticus*, and *Lactobacillus lactis*, was introduced into the milk. The starter was inoculated as a 50-unit colony (UC) in one liter of milk. Cheese wheels (*n* = 6) were produced: *n* = 3 uninoculated cheese wheels (control, B) as the control, and *n* = 3 cheese wheels inoculated with *L. innocua* (L) strains (see below). *L. innocua* was used instead of *L. monocytogenes* for safety purposes, as it is considered a non-pathogenic surrogate sharing similar ecological characteristics with *L. monocytogenes* [48].

### 2.2. Contamination of Milk

In order to experimentally contaminate the milk used for cheese making with *L. innocua*, an inoculum was prepared in accordance with the past guidelines “EURL Technical Guidance Document for Conducting Shelf-life Studies on *Listeria monocytogenes* in Ready-to-Eat Food” [49]. *L. innocua* was used as a non-pathogenic proxy of *L. monocytogenes*. Note that *L. innocua* is very similar to *L. monocytogenes*, except for a few enzymatic processes: hemolysis (negative), arylamidase (positive), and phosphoinositide phospholipase C (negative). Each type of cheese was produced in triplicate. The cheesemaking activity was performed on two consecutive days. In order to reduce the strain-related biases, we inoculated milk with three different *Listeria* strains: *L. innocua* ATCC 33090, *L. innocua* 30/9/97 D, and *L. innocua* 3/10/97 C. All these strains were stored at the Istituto Zooprofilattico Sperimentale delle Venezie (IZSVe) in cryobeads at −80 °C, with preservative medium (Copan Diagnostics, Murrieta, CA, USA) and are in use for challenging tests purposes (EN ISO 20976-1 [50]). The three *L. innocua* strains were mixed together to achieve a final concentration of 7 × 10^2^ CFU/200 L in the starting milk for cheesemaking. In detail, two subsequent subcultures of each *L. innocua* strain were prepared, starting from the cryobeads. The first subculture was prepared by transferring a cryobead into 9 mL of Brain Heart Infusion (BHI) broth and incubating at 37 °C for 15–18 h. The second subculture was obtained by transferring 0.1 mL of the first subculture into 9 mL of fresh BHI and incubating the broth at 20 °C for 72 h. After 15–18 h, the three *Listeria* cultures were at OD600 = 1. The day before the milk inoculation, 1 mL was taken from each of the three broth cultures and diluted in 9 mL of physiological saline. After that, 4 mL from each of the three diluted broth cultures were taken and mixed in a single test tube to obtain a bacterial suspension of 12 mL, with a final concentration of 1.4 × 10^8^ CFU/mL. To quantify the effective bacterial load in the inoculum suspension, appropriate 10-fold dilutions were prepared; 100 μL of each dilution was seeded onto ALOA (Agar Listeria by Ottaviani and Agosti) plates and counted after incubation for 48 h at 37 °C. The entire volume of the bacterial suspension was used to inoculate 2 L of milk. This milk was then made up to the final volume of 200 L. The expected final concentration of *L. innocua* in the starting milk was 7.0 × 10^2^ CFU/200 L.

### 2.3. Sampling of Cheese Wheels

The prepared cheese wheels were stored for 60 days in a refrigerated aging cell (Salumajo 400, O. Majolo, Mezzavia, Italy), with 85% relative humidity and temperature set at 8 °C. Samples were taken for metataxonomic and metabolomic analyses at the following time points: T0 = day 0, T1 = 7 days post-inoculation (dpi), T2 = 21 dpi, T3 = 28 dpi, T4 = 35 dpi, T5 = 49 dpi, T6 = 56 dpi, T7 = 60 dpi. The samples were collected by core drilling from the cheese wheel sides. After the drilling, the spaces left empty inside the wheels were sealed with a sterile food putty specially formulated for the conservation of cheese wheels (Parafluid stucco plastic, CIP, Cremona, Italy).

### 2.4. Microbiological Analyses

For the microbiological analyses, two corings were carried out, and at each sampling point, about 30 g of cheese was taken. A total of 10 g of cheese was weighed into a sterile stomacher bag together with 90 mL of tryptone solution (TS) for the enumeration of mesophilic microorganisms and lactic acid bacteria, and 10 g of cheese with 90 mL of buffered peptone water (BPW) was used for the enumeration of *L. innocua*.

#### 2.4.1. Quantification of Mesophilic Microorganisms

The quantification of the total mesophilic bacterial load was performed using 10 g of cheese homogenized in TS and in accordance with ISO 4833-1:2013 [51]; 1 mL volumes of homogenized cheese in TS were seeded in appropriate 10-fold dilutions (prepared in TS) onto plate count agar (PCA) plates. After incubation at 30 °C for 72 h, the colonies were counted. Plates containing 10 to 300 colonies were taken into consideration (ISO 4833-1:2013).

#### 2.4.2. Quantification of Lactic Acid Bacteria (LAB)

The quantification of LAB at 30 °C in aerobiosis was performed by seeding 1 mL of homogenized cheese in TS, at appropriate 10-fold dilutions in TS, onto double-solidified MRS (De Man, Rogosa, and Sharpe) agar medium. The plates were incubated at 30 °C for 72 h in aerobiosis, and then typical lactobacilli colonies were counted. Plates containing 10 to 300 colonies were taken into consideration (ISO 7218:2007 [52]).

#### 2.4.3. Quantification of *L. innocua*

For the determination of *L. innocua*, appropriate 10-fold dilutions of the homogenized cheese in BPW, prepared using buffered peptone water for *Listeria* (APTL), were cultured on ALOA plates. The plates were incubated at 37 °C for 18–24 h, and typical colonies were counted. The limit of detection of the method was <10 CFU/g.

### 2.5. RNA Extraction and Retrotranscription

For RNA extraction, the cheese wheels were sampled as described before (see Section 2.4) to collect about 15 g from each wheel at each time point. Cheese from each time point was stored in RNAlater^®^ (SigmaAldrich, St. Louis, MO, USA) before RNA extraction. Fifty mg of cheese was taken from each sample and used for total RNA extraction. Each sample was extracted in duplicate. RNA extraction was performed with Power Lyzer Ultraclean Tissue&Cells RNA Isolation Kit (MOBIO Laboratories Inc., London, UK) following the manufacturer’s instructions.

Ceramic beads were added to the sample together with 600 μL of TR1, containing 10 μL βME per mL of TR1 solution, to resuspend the samples. Samples were kept on ice and homogenized with Disruptor Genie (Scientific Industries, Bohemia, NY, USA) by stirring for 1 min three times, with ice cooling in between. Samples were then centrifuged (13,000× *g*, 1 min) to collect the supernatants. TR2 (600 μL) was added to supernatants, transferred into new tubes, and mixed by pipetting. After that, first, 600 μL were transferred into Spin Filter tubes and centrifuged (11,000× *g*, 1 min). This step was repeated for the remaining volume. Tubes were then washed with 500 μL of TR3 (WB Wash Buffer) and centrifuged (11,000× *g*, 1 min), then washed again two times with 500 μL of TR4 (RW Remove Wash) followed by a centrifugation step (11,000× *g*, 1 min). A final centrifugation step (13,000× *g*, 2 min) was performed to remove ethanol traces from the Spin Filter tubes. RNA was then eluted with 40 μL of TR5 (RNase-free water) after incubation at room temperature for 1 min and centrifugation (11,000× *g*, 1 min). After RNA extraction, TURBO™DNAse (Life Technologies, Carlsbad, CA, USA) was added to each sample to remove possible DNA contaminations. RNA was conserved at −80 °C for subsequent analyses. Retrotranscription of RNA was performed using SuperScript™IIReverseTranscriptase (Invitrogen, Waltham, MA, USA) following the instructions of the manufacturer.

### 2.6. rDNA16S Sequencing

The resultant templates were submitted to the amplification of the V3–V4 hypervariable regions of the 16S rRNA gene. The 16S library was prepared according to the Illumina 16S Metagenomic sequencing Library Preparation protocol, using the primers Bact341F and Bact785R (Fwd: CCTACGGGNGGCWGCAG and Rev: GACTACHVGGGTATCTAATCC) previously described by Klindworth et al. (2013) [53], using the Nextera XT DNA Library Prep kit (Illumina, San Diego, CA, USA). The amplification check was performed using 2% TAE agarose gel electrophoresis with the aim of identifying DNA fragments 550 bp in length. Libraries were checked for both concentration and quality using Qubit and 2200 TapeStation (Agilent, Santa Clara, CA, USA), respectively. Samples were equimolarity pooled, and sequencing was performed with an Illumina MiSeq platform with a MiSeq 600V3 cartridge (600 cycles, 2 × 300 bp, paired-end reads). After sequencing, data were submitted to a quality control procedure using the FastQC tool(version 0.1.4) [54]. All subsequent steps were performed using Quantitative Insights Into Microbial Ecology 2 (QIIME2) version 2020.2 pipeline [55]. Raw sequence data were screened, trimmed, and denoised with the DADA2 (version 1.28) open-source software package [56] and then quality filtered. Operational taxonomic units (OTUs) were defined as sequences with at least 97% similarity, and taxonomy was assigned by the SILVA database [57]. The rarefaction depth was based on the lowest read depth of samples. Graphical output was obtained by the ggplot package of RStudio software [58].

### 2.7. Microbial Communities’ Analyses

Statistical analyses were conducted with the phyloseq package of RStudio software. For alpha-diversity measures (richness, Shannon and Pielou’s Indexes), the count table was normalized using the Geometric Mean of Pairwise Rations method (GMPR) package (version 0.1.3). The non-parametric Kruskal–Wallis test was used to compare alpha diversity. If significant, the pairwise comparison Wilcoxon test was performed with an adjusted *p*-value (padj-value) for multiple comparisons using the false discovery rate (FDR) method. Beta-diversity was evaluated with Bray–Curtis distance and visualized in a Principal Coordinates Analysis (PCoA) plot. The PERMANOVA test was used to compare beta-diversity parameters between groups. Tests were considered significant if *p*-value < 0.05.

### 2.8. DART-HRMS Analysis

Control cheese and *L. innocua*-inoculated cheese were analyzed at different times from T0 to T7, as described in Section 2.3 above. Two sample extraction procedures were used. In the first one, 0.5 g of cheese was suspended in 5 mL of a solution of water and methanol (20:80 *v*/*v*) (MilliQ water and methanol HPLC-grade with 99.9% purity, from VWR International, Radnor, PA, USA), vortexed, sonicated for 15 min, and centrifuged at 12,000× *g* for 5 min. In the second protocol, 0.5 g of cheese was suspended in 5 mL of ethyl acetate (99.9% purity, Carlo Erba Reagents, Cornaredo, Italy), vortexed, sonicated for 15 min, and centrifuged 12,000× *g* for 5 min. The chemical profile of the extracts was acquired using a DART SVP 100 ion source (IonSense, Saugus, MA, USA) coupled with an Exactive Orbitrap (Thermo Fisher Scientific, Waltham, MA, USA). The DART source was coupled with a Dip-it(R) autosampler (IonSense, Saugus, MA, USA). Glass capillary rods were inserted into a holder of the autosampler, and then 5 µL of each extracted sample was spotted on each glass capillary rod. A vapor interface was positioned between the ion source and the mass spectrometer. The optimized settings of the source and the mass spectrometer are reported in the Supplementary Material (Tables S1 and S2). In positive ion mode, a vial with an aqueous solution of 25% ammonia was positioned below the DART gun exit, working as a dopant to facilitate and stabilize the formation of [M + NH4]^+^ ions. Three instrumental repetitions of each sample extract were acquired to improve repeatability. Prior to statistical analysis, the spectra of the 4 datasets (2 extraction solvents per 2 ion modes) were converted to .csv files with Rstudio 3.6.1 software (RStudio Team, 2016; RStudio Integrated Development for R; RStudio, Inc., Boston, MA, USA). The data in these files were submitted to statistical analysis using the online platform MetaboAnalyst 5.0 (www.metaboanalyst.ca (accessed on 2 May 2024)).

### 2.9. Data Processing and Statistical Analysis

The 16S rRNA sequencing data and metabolomics data were statistically analyzed using the MetaboAnalyst 5.0 web platform (www.metaboanalyst.ca (accessed on 2 May 2024)) and Rstudio 3.6.1 software. The DART-HRMS data were aligned, and isotopes were removed using the R script. The three instrumental spectral repetitions, nor the spectra of the different wheels of inoculated cheese, were averaged to enable us to catch the heterogeneity of the extracts. Averaging the replicates in class discriminations is usually discouraged in non-targeted approaches, as the averaging could lead to the loss of valuable metabolic information linked to intra- and inter-sample variability [59]. Then, the signals of each spectrum were normalized by sum, and each *m*/*z* feature was scaled by Pareto. A low-level data fusion of the (+/−)DART-HRMS data (2 extraction solvents per 2 ion modes) was performed. In a low-level data fusion, the raw signals obtained from the four data matrices were merged and then processed as a unique fingerprint of the samples [60]. Partial least squares-discriminant analysis (PLS-DA) of the merged datasets (2 extraction solvents per 2 ion modes) of control and *L. innocua*-inoculated cheese was performed to visualize the differences between the fingerprints. Afterward, a PLS-DA was also performed to evaluate the possible clustering of *L. innocua* inoculated cheese at different time points. Two-way ANOVA was carried out on merged (+/−)DART-HRMS data, with *Listeria innocua* inoculation and time as the factors. The resultant statistical significance was set at adjusted *p*-value < 0.05 using the false discovery rate (FDR) criterion. Finally, microbiota and metabolomic datasets were merged, and the MixOmics package version 6.10.x [44] was implemented in R software (Rstudio 4.0.2) to determine the possible correlation between the abundances of OTUs from the 16S rRNA gene sequencing and the intensity of metabolites selected by volcano plot. To this aim, the merged multiomic dataset was submitted to sparse partial least squares discriminant analysis (sPLS-DA), and sPLS-DA correlation networking analysis was performed. Note that the conventional rule for interpreting the degree of a correlation expressed by a correlation coefficient is the following: 0.9 to 1, very high positive correlation (−0.9 to −1, very high negative); from 0.7 to 0.9, high positive correlation (−0.7 to −0.9, high negative); from 0.5 to 0.7, moderate positive correlation (−0.5 to −0.7, moderate negative) [61]. Correlation coefficients ranging between 0.5 and −0.5 are considered weak to negligible. All the significant metabolomic features (*m*/*z* values) teased out by statistical analysis were tentatively annotated using high mass accuracy and a library search on the metabolome database (https://hmdb.ca (accessed on 10 May 2024)) and (https://foodb.ca (accessed on 10 May 2024)).

## 3. Results

**Figure 2:**
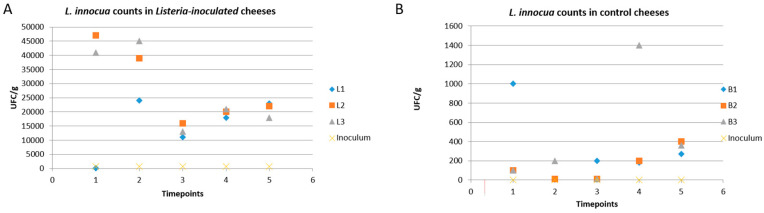
*Listeria*-inoculated (**A**) and *L. innocua* counts in control cheeses (**B**). Counts are reported for three uninoculated cheese wheels (control cheeses, panel (**B**)) indicated as B1, B2, and B3, and for three cheese wheels inoculated with *L. innocua* (*Listeria*-inoculated cheeses, panel (**A**)) indicated as L1, L2, and L3. Counts are reported as CFU/g, while data were collected at different time points (T0 to T5) that correspond to 0 to 49 days post-inoculation, as explained in the main text (see Section 2.2 Contamination of Milk).

The resident bacteria counts were very high during both the cheesemaking and cheese-ripening phases, with an increase in *L. innocua* at T1 and T2 of the ripening process and subsequent inhibition of its growth at T3 [23,62] (Figure 2).

As expected, *L. innocua* was not detected at countable levels in the control cheeses.

**Figure 3:**
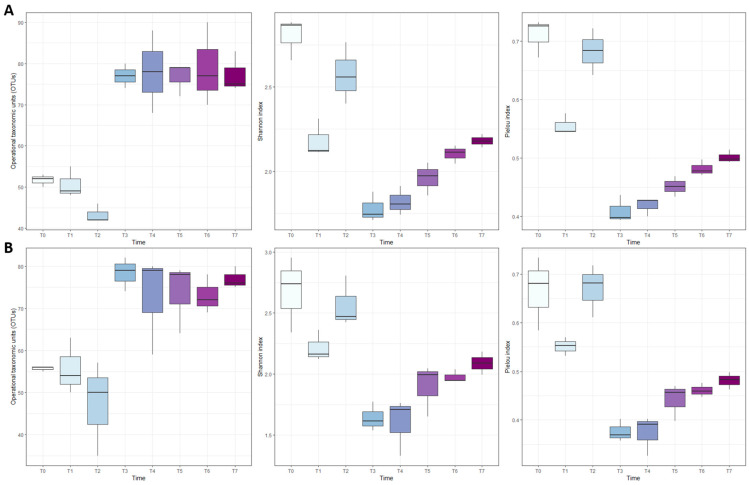
Boxplots of the number of identified Operational Taxonomic Units (OTUs) and alpha-diversity (Shannon and Pielou indices) for control cheese (panel (**A**)) and *L. innocua*-inoculated cheese (panel (**B**)). Time = sampling point. Different colors are used for each sampling point.

The diversity analysis showed the same trend for the microbial community both in the control cheeses (Figure 3A) and in inoculated cheeses (Figure 3B). In both types of cheese, the microbial community underwent a shift in richness and evenness between T2 and T3. In particular, the number of OTUs increased after T2, while Shannon’s index and Pielou’s index decreased after this time point.

**Figure 4:**
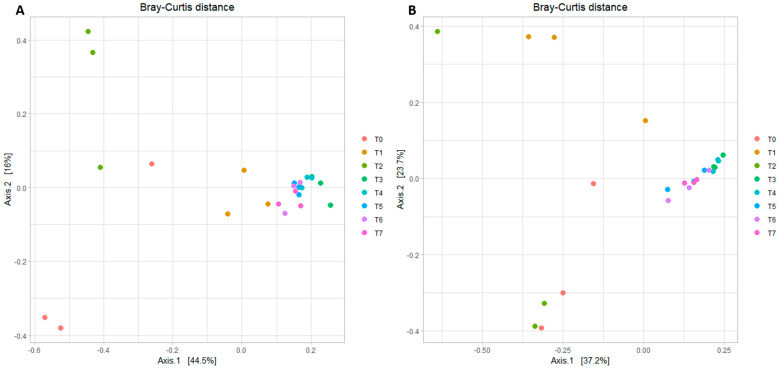
Principal coordinates analysis (PCoA) plot of beta diversity for each cheese sampling point (T0 to T7). Samples from control cheese are shown in Panel (**A**), while samples from *L. innocua*-inoculated cheese are reported in Panel (**B**). Each point represents the microbial community of a sample. Within each plot, samples with similar microbiota composition tend to be in the same area of the plot, while points far apart from each other represent samples with dissimilar microbiota. Similar conclusions can be drawn from the results of beta-diversity analyses.

In Figure 4 it is clear that in both types of cheese (control and inoculated), the microbial community structure converged during the ripening period. Samples from T0, T1, and T2 were more distant in space on the plots than samples collected later during ripening.

**Figure 5:**
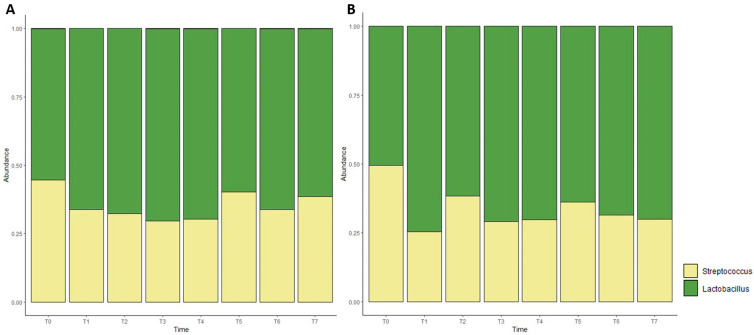
Stacked bar charts showing relative abundance (%) of microbial communities, annotated to the taxonomic level of genus, derived from control cheese (**A**) and *L. innocua*-inoculated cheese (**B**).

To display the proportion of different taxa at the genus level, bar plots were generated based on the relative abundance of taxa (Figure 5). The results showed that the most representative genera of bacteria in the samples of the entire dataset were *Lactobacillus* and *Streptococcus*.

**Figure 6:**
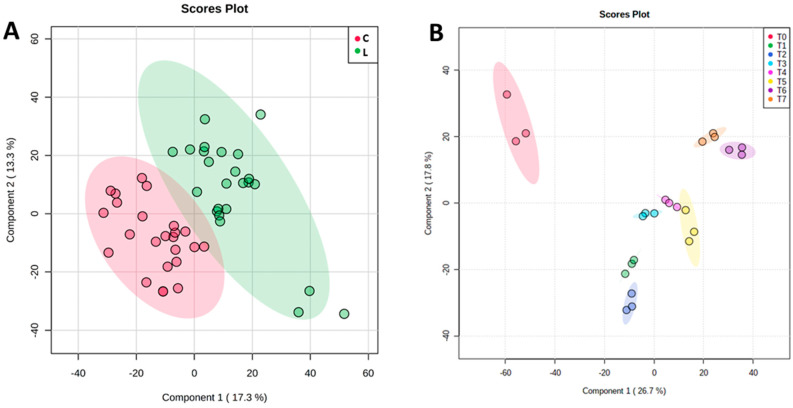
Differences in the metabolic profile of control cheese and cheese inoculated with *L. innocua*. (**A**) Partial least squared discriminant analysis (PLS-DA) score plot illustrating the variation of metabolic profile between control cheese (C, in red) and cheese inoculated with *L. innocua* (L, in green). (**B**) PLS-DA score plot illustrating the time-related (time points T0 to T7) metabolic differences in ripening cheese inoculated with *L. innocua*.

On the other hand, comparing the metabolic fingerprinting of control cheese with that of *L. innocua*-inoculated cheese, a clear differentiation can be observed (Figure 6). The PLS-DA score plot shows a well-defined clustering of samples from the two cheese types in the graphical space defined by the first and second principal components, C1 and C2, which explained 17.3% and 13.3% of the total variance of the model, respectively (Figure 5). The results highlight DART-HRMS’s ability to capture the high variability of the two groups based on the differences in their metabolic profile likely caused by the presence of *L. innocua*. The PLS-DA teased out the ions that contribute significantly to the discrimination of *L. innocua*-inoculated cheese (Table 1).

**Figure 7:**
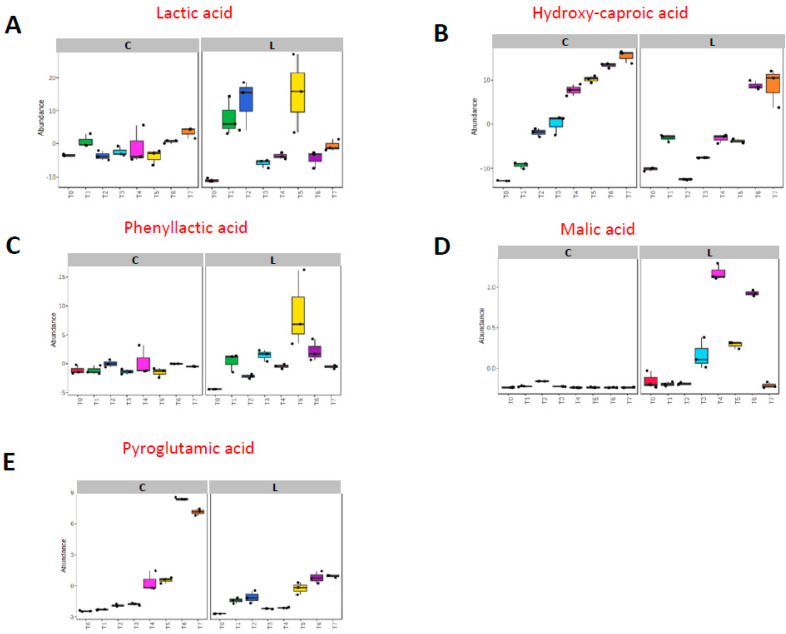
Most significant time-related metabolic changes in control (C) and *L. innocua*-inoculated (L) cheese at time points T0 to T7 (*p*-value < 0.05). The 2-way ANOVA (adjusted *p*-value < 0.05) captured six important metabolites: (**A**) lactic acid; (**B**) hydroxy-caproic acid; (**C**) phenyllactic acid; (**D**) malic acid; (**E**) pyroglutamic acid. The amounts of these metabolites were significantly different at some time points (T0 to T7) in control and inoculated cheeses. Different colors represent the different time points. Data are presented as mean (+ symbol), median (center line), interquartile range (shaded area), and range (error bars).

**Figure 8:**
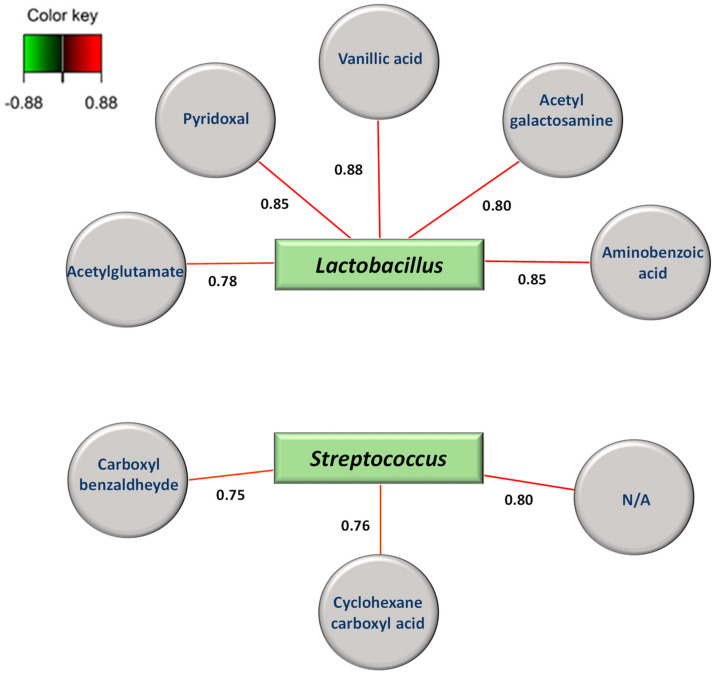
Spares partial least squared discriminant analysis (sPLS-DA) correlations between dominant bacterial genera and metabolites. The model allowed visualization of a casual correlation between bacterial genera and metabolites in *L. innocua*-inoculated cheese.

Moreover, good discrimination of the *L. innocua*-inoculated cheese collected at different ripening times was also achieved. PLS-DA produced excellent clusterization of the eight groups by the first and second principal components, C1 and C2, which explained 46.2% and 14.9% of the total variance of the model, respectively (Figure 6B). This result indicates that the metabolic profile of *L. innocua*-inoculated cheese also changed with time. A 2-way ANOVA of the merged (+/−)DART-HRMS data allowed us to capture both the overall metabolic differences due to the *Listeria innocua* inoculation and how the metabolites differed at the different time points. The metabolites (organic acids, hydroxyl fatty acids, and antimicrobial compounds) that contributed the most to discriminating the *L. innocua*-inoculated cheeses from the control cheeses and time points are shown in Figure 7 and detailed in Table 1. We observed a higher abundance of lactic acid at T1, T2, and T5 than at other time points in the inoculated cheese than in the control cheese (Figure 7A), and a higher level of hydroxy-caproic acid was captured in the control cheese than in the inoculated cheese (Figure 7B). In the *L. innocua*-inoculated cheese, the 2-way ANOVA revealed a high level of phenyllactic acid at T1, T3, and T5 (Figure 7C), while more malic acid was mainly seen at T3 and after (Figure 7D). A higher level of pyroglutamic acid was found in the control at T4 and T7 than in other time points (Figure 7E). Moreover, an overall higher abundance of pyroglutamic acid was observed in the control cheese than in the inoculated cheese (Figure 7E). These metabolites are highlighted in bold in Table 1. Figure 8 illustrates the correlations between the dominant bacterial genera in the inoculated cheese and metabolites. We observed positive strong correlations between increased levels of *Lactobacillus* spp. and acetylglutamate, pyroxidal, vanillic acid, acetylgalactosamine, and aminobenzoic acid. On the other hand, enhanced levels of *Streptococcus* spp. were positively correlated with carboxylbenzaldheyde and cyclohexanecarboxylic acid.

## 4. Discussion

In this study, a combined strategy based on non-targeted metabolomics and metagenomics is applied to elucidate the interaction of resident microbiota with *L. innocua* in cheese. Analysis of the microbial community revealed that inoculation of *L. innocua* did not alter the composition of the microbial community during the ripening period of cheese. The trend of the microbial community development (in terms of alpha-diversity, beta-diversity, and composition) throughout the cheese ripening was the same both in the presence and absence of this microorganism.

It is worth noticing the sudden change in metrics related to alpha and beta diversity between T2 and T3. However, this variation could not be fully explained in terms of cheese’s microbial composition at the genus level. The reason for the change could be linked to the physico-chemical variations that occur during the ripening process that promoted the growth of some bacterial species over others between T2 and T3. In accordance with the metataxonomic observations, very distinct changes in metabolic profiles were also observed between T2 and T3, with an increase in malic acid, a decrease in lactic acid, and fluctuations in hydroxy-caproic acid and phenyllactic acid.

Through the changes in their own metabolism and the subsequent release of antimicrobial molecules, the lactic acid bacteria impact the growth of pathogens [70]. The dynamics of variation of the resident microbiota in control and *L. innocua*-inoculated cheese observed in our study are consistent with the findings of other research involving raw milk in the production of Gouda cheese [71]. The authors claimed that in the first phase of cheese production, the microbial populations were dominated by the starter microorganisms, whose main technological action was carried out with the lowering of the milk pH; during the maturation phase, the counts of these bacteria slightly decreased, and the bacteria were involved in other important enzymatic reactions such as lipolysis, proteolysis, and production of flavor compounds. Furthermore, the authors stated that the inoculum of *Listeria monocytogenes* had no inhibitory effect on the relative abundance of starter populations, nor vice versa [71].

Recently, several antifungal and antibacterial compounds (cyclic dipeptides, phenyllactic acid, hydroxyphenyllactic acid), small organic acids (lactic, acetic, formic, citric, malic, and succinic acid), and 3-hydroxylated fatty acids have been isolated from lactic acid bacteria interacting with pathogens [72]. Our strategy of merging multiple metabolic profiles (2 extraction solvents per 2 ion modes) instead of tackling them separately revealed differences in the relative intensities of some chemical compounds between control and *L. innocua*-inoculated cheese that are supported by the literature. Similar to our results, Fuochi et al. [11] found a high concentration of lactic acid in the supernatants of all lactic acid bacteria strains interacting with the pathogen *Legionella*. The fermentation of lactose to lactic acid by lactic acid bacteria is an essential primary reaction in the production of cheese [73]. Recent studies showed that lactic acid alone has a weak inhibitory effect against pathogens, suggesting that it acts synergically with other metabolites [13,74].

Interestingly, at time points T1, T3, and T5, a greater relative abundance of phenyllactic acid was observed in *L. innocua*-inoculated cheese. Phenyllactic acid is recognized as the major factor responsible for the antifungal activity of *Lactobacillus* spp. [13,68,75]. The inhibitory properties of phenyllactic acid have been demonstrated against several fungal species isolated from bakery products [76] and some bacterial species such as *Listeria* spp. [77], *Staphylococcus aureus*, and *Enterococcus faecalis* [77]. Phenyllactic acid is a metabolite derived from the catabolism of amino acids. Its structural similarity to amino acids suggests that phenyllactic acid is formed by the transamination route via a keto-acid, which is reduced by a hydroxyacid dehydrogenase [12]. Lavermicocca et al. [75] hypothesized that, to avoid intracellular accumulation of phenylalanine, this amino acid may be hydroxylated to tyrosine or transaminated to phenylpyruvic acid, which is further metabolized to phenylactic and hydroxyphenyllactic acids. They supposed that phenyllactic acid is excreted in copious amounts during the growth of *L. plantarum* to avoid the intracellular accumulation of phenylalanine. An interesting study proposed the addition of phenyllactic acid to prevent the growth of *Listeria* in milk and cheese [77]. In accordance with our results, Park et al. [78] recently identified 2-hydroxyisocaproic acid production from lactic acid bacteria in kimchi ripening, suggesting its use as an indicator for foods fermented with lactic acid bacteria. Phenyllactic acid and 2-hydroxyisocaproic acid, together with other compounds, have previously been found in *L. reuteri* supernatant able to inhibit *Microsporum canis*, *Microsporum gypseum*, *Epidermophyton floccosum* [79], *Fusarium culmorum*, and *Aspergillus fumigatus* [80]. The ability of malic acid to inhibit *L. monocytogenes* has also been previously confirmed in laboratory media [81,82]. Specifically, its inhibition effect against *L. monocytogenes* was demonstrated using malic acid-impregnated whey protein films.

Considering all the literature studies mentioned above, we could hypothesize that the increased production of antimicrobial compounds in *Listeria*-inoculated cheese could be due to the response of the resident microbiota to the presence of the pathogen.

Similarly to our outcomes, Peng et al. [83] claimed that *Lactobacillus* release antimicrobial molecules, like small organic acids, hydrogen peroxide, polypeptides, and lipid peroxides, to outcompete undesirable microorganisms in a time-dependent manner. The metabolite pyroglutamic acid has been previously quantified in many cheese varieties in ripened Italian cheeses produced with thermophilic lactic acid bacteria as starters and was proposed as a marker of the ripening process [84].

Finally, in the present study, positive correlations were encountered between *Lactobacillus* and acetylglutamate, pyroxidal, vanillic acid, acetylgalactosamine, and aminobenzoic acid. While pyridoxal is one of the compounds responsible for the modifications shown by volatile metabolic fingerprinting and induced by bacterial metabolism in cheese over time [85], aminobenzoic acid is an essential compound in *Lactobacillus* involved in folate metabolism [86]. *Lactobacillus* has been previously reported to produce vanillic acid, which showed moderate antifungal activity in vitro during microbial degradation of the cinnamic acid derivates present in the silage used to feed the cows, possibly via the intermediate formation of hydrocinnamic acid [87]. Flavor production in fermented food is often inseparable from the action of microorganisms [88]. Among bacteria, *Lactobacillus* is widely reported to play an important role in the determination of food aroma [89]. Finally, acetylgalactosamine is involved in sugar catabolism and the biosynthesis and engineering of exopolysaccharide production in lactic acid bacteria [90].

Our correlation network also positively associated enhanced levels of *Streptococcus* with carboxylbenzaldheyde and cyclohexanecarboxylic acid. Interestingly, cyclohexanecarboxylic acid was previously detected during the storage of milk fermented with pure cultures of *Streptococcus thermophilus* and mixed cultures of *Lactobacillus delbrueckii* spp. *bulgaricus* and *Streptococcus thermophilus* [91].

## 5. Conclusions

Integrating multiple molecular approaches, including high throughput microbial community profiling, non-targeted metabolic profiling could provide a deeper insight into food microbial ecology at a systems level. This work draws a detailed picture of the microbial community dynamics and the metabolic differences in *Listeria*-inoculated cheese and control cheese. In detail, the diversity analysis showed the same microbial diversity trend in both the control cheese and the inoculated cheese, while the taxonomic analysis highlighted the most representative genera of bacteria in both the control and inoculated cheese: *Lactobacillus* and *Streptococcus*.

On the other hand, DART-HRMS revealed that the complex interactions between resident microbiota in *Listeria*-inoculated cheese were governed by continuously changing chemical signals. Variations in relative abundances of small organic acids, hydroxyl fatty acids, and antimicrobial compounds, including pyroglutamic acid, hydroxy-isocaproic acid, malic acid, phenyllactic acid, and lactic acid, were observed during cheese ripening in *Listeria*-inoculated cheeses. Finally, some correlations between the resident microbiota and metabolic expression were established. In cheese experimentally inoculated with *Listeria*, the resident *Streptococcus* was significantly correlated with the volatile compounds carboxylbenzaldheyde and cyclohexanecarboxylic acid, while *Lactobacillus* was positively correlated with some volatile and flavor compounds (cyclohexanecarboxylic acid, pyroxidal, aminobenzoic acid, and vanillic acid) involved in cheese sensory properties. Therefore, we determined the metabolic markers that characterize a raw milk cheese inoculated with *L. innocua*, the changes in these markers with the ripening time, and the positive correlation of flavor and volatile compounds with the resident microbiota.

Capitalizing on the potential complementarities of metataxonomic and metabolomic data, the results of this study may open up a new avenue through the integration of microbial ecology data and metabolomics in the effective control of undesirable microorganisms in food matrices. This multi-omics approach could suggest innovative food safety strategies based on the enhanced control and management of undesirable microorganisms by means of strain selection for microbial inoculation in raw food matrices and the addition of specific antimicrobial metabolites (such as hydroxy-isocaproic acid, phenyllactic acid, and lactic acid) to prevent the growth of undesirable microorganisms.

Finally, we demonstrated the effective usefulness of ambient mass spectrometry by DART-HRMS in the assessment of metabolic changes linked to microbial community dynamics.

## Figures and Tables

**Table 1:** Significant metabolites teased out by PLS-DA that differentiate a cheese inoculated with *L. innocua* from a control. The experimental mass/ratio (*m*/*z*) values, the theoretical mass/ratio (*m*/*z* theor), the error in ppm, the assignment, the type of ion, the time point of observation, the instrument ion mode, the extraction procedure, the elemental formula, and the literature references are reported. Ions highlighted in bold showed significant changes in abundance over time, according to 2-way ANOVA.

*m*/*z*	*m*/*z* Theor	Error (ppm)	Formula	Type of Ion	Extraction and Ion Mode	Tentative Assignment	References
89.0242	89.0244	−2.25	C_3_H_6_O_3_	[M−H]^−^	MeOH:H_2_O (80:20 *v*/*v*)	**Lactic acid**	[11,63]
111.0078	111.0082	−3.6	C_5_H_4_O_3_	[M−H]^−^	(+) Pure EtAc	Mesaconic acid	
128.035	128.0353	−2.34	C_5_H_7_NO_3_	[M+H]^+^	(+) MeOH:H_2_O (80:20 *v*/*v*)	**Pyroglutamic acid or oxoproline**	[45]
132.1015	132.1013	−1.5	C_6_H_12_O_3_	[M+NH_4_−H_2_O]^+^	(+) MeOH:H_2_O (80:20 *v*/*v*)	**Hydroxy-isocaproic acid**	[11,12,64,65,66]
133.0137	133.0142	−3.76	C_4_H_6_O_5_	[M−H]^−^	(−) MeOH:H_2_O (80:20 *v*/*v*)	**Malic acid**	[14,66,67]
165.0554	165.0557	−1.81	C_9_H_10_O_3_	[M−H]^−^	(−) MeOH:H_2_O (80:20 *v*/*v*) (−) Pure EtAc	**Phenyllactic acid**	[13,64,68]
191.0187	191.0197	−5.2	C_6_H_8_O_7_	[M−H]^−^	(−) MeOH:H_2_O (80:20 *v*/*v*)	Citric acid	
210.0602	210.0608	−2.85	C_6_H8O_7_	[M+NH_4_]^+^	(+) MeOH:H_2_O (80:20 *v*/*v*)	Citric acid	[69]
255.2329	255.2330	−0.39	C_16_H_32_O_2_	[M−H]^−^	(−) Pure EtAc	Palmitic acid	
256.2625					(+) MeOH:H_2_O (80:20 *v*/*v*)	N/A	
281.2485	281.2486	0.35	C_18_H_34_O_2_	[M−H]^−^	(−) Pure EtAc	Vaccenic acid/oleic acid	
296.2573	296.2584	3.7	C_18_H_30_O_2_	[M+NH_4_]^+^	(+)MeOH:H_2_O (80:20 *v*/*v*)	alpha- and/or gamma-linolenic acid	
305.1743	305.1749	1.9	C_11_H_23_N_5_O_3_S	[M+NH_4_−H_2_O]^+^	(+) Pure EtAc	Methionyl-Arginine	
313.2731	313.2743	−3.8	C_19_H_38_O_4_	[M+H−H_2_O]^+^	(+) Pure EtAc	MG (20:2)	
316.2113					(+) Pure EtAc	N/A	
327.2521	327.2530	−2.75	C_19_H_36_O_5_	[M+H−H_2_O]^+^	(+) Pure EtAc	DG (16:0)	
339.2327	339.2330	0.88	C_23_H_32_O_2_	[M−H]^−^	(−) Pure EtAc	N/A	
355.2834	355.2843	2.53	C_21_H_38_O_4_	[M+H]^+^	(+) Pure EtAc	MG (18:2)	
383.3149	383.3156	−1.82	C_23_H_42_O_4_	[M+H]^+^	(+) Pure EtAc	MG (20:2)	
411.3461	411.3469	1.94	C_25_H_48_O_4_	[M+H]^+^	(+) Pure EtAc	MG (22:1)	
523.4706	523.4727	4.011	C_33_H_64_O_5_	[M+H−H_2_O]^+^	(+) Pure EtAc	DG (30:0)	

N/A: non assigned; MG: Monoacylglycerols; DG: Diacylglycerols.

## Data Availability

The original contributions presented in the study are included in the article/Supplementary Material, further inquiries can be directed to the corresponding author.

## Supplementary Materials

The following supporting information can be downloaded at: https://www.mdpi.com/article/10.3390/foods13121912/s1, Table S1: Instrumental settings of DART source in positive and negative ion mode; Table S2: Instrumental settings of the orbitrap analyzer in positive and negative ion mode.
