# Supplementary material for: A Snapshot, Using a Multi-Omic Approach, of the Metabolic Cross-Talk and the Dynamics of the Resident Microbiota in Ripening Cheese Inoculated with Listeria innocua
Source: Foods. 2024 Jun 18;13(12):1912. doi: 10.3390/foods13121912 (PMC11203185; doi:10.3390/foods13121912)
Supplement: Supplementary file 1 [file foods-13-01912-s001.zip › foods-3048609-supplementary.pdf]

A Snapshot, Using a Multi-Omic Approach, of the Metabolic Cross-Talk and the Dynamics of the Resident Microbiota in Ripening Cheese Inoculated with *Listeria innocua*

Alessandra Tata [1,*,†], Andrea Massaro [1,†], Brunella Miano [1], Sara Petrin [2], Pietro Antonelli [2], Arianna Peruzzo [2,3], Alessandra Pezzuto [4], Michela Favretti [4], Marco Bragolusi [1], Carmela Zacometti [1], Carmen Losasso [2,‡] and Roberto Piro [1,‡]

[1] Laboratorio di Chimica Sperimentale, Istituto Zooprofilattico Sperimentale delle Venezie, Viale Fiume 78, 36100 Vicenza, Italy
[2] Laboratory of Microbial Ecology and Genomics, Istituto Zooprofilattico Sperimentale delle Venezie, Viale dell'Università, 35020 Legnaro, Italy
[3] PhD National Programme in One Health Approaches to Infectious Diseases and Life Science Research, Department of Public Health, Experimental and Forensic Medicine, University of Pavia, 27100 Pavia, Italy
[4] Laboratory of Hygiene and Safety of the Food Chain, Istituto Zooprofilattico Sperimentale delle Venezie, Viale dell'Università, 35020 Legnaro, Italy
* Corresponding author: atata@izsvenezie.it
† These authors contributed equally to this work.
‡ These authors contributed equally to this work.

Supplementary material

Table S1. Instrumental settings of DART source in positive and negative ion mode

**DART**	
Temperature	300 °C°
Grid Voltage	250V
Helium flow	4.26 L/min

Table S2. Instrumental settings of the orbitrap analyzer in positive and negative ion mode

**Orbitrap**	
Mass Range	da 75 a 1125 *m/z*
Resolution	70,000 FWHM at 200 m/z
Capillary temperature	250 °C
S-lens RF level	55
CID	0 eV
Maximum injection time	10 ms
